# Circulating levels of growth hormone in postural orthostatic tachycardia syndrome

**DOI:** 10.1038/s41598-021-87983-5

**Published:** 2021-04-21

**Authors:** Madeleine Johansson, Fabrizio Ricci, Janin Schulte, Margaretha Persson, Olle Melander, Richard Sutton, Viktor Hamrefors, Artur Fedorowski

**Affiliations:** 1 Department of Clinical Sciences, Faculty of Medicine, Clinical Research Center, Lund University, Box 50332, 20213 Malmö, Sweden; 2 Department of Cardiology, Skåne University Hospital, Malmö, Sweden; 3 Department of Neuroscience, Imaging and Clinical Sciences, Institute for Advanced Biomedical Technologies, “G.d’Annunzio” University, Chieti, Italy; 4 Casa di Cura Villa Serena, Cittá Sant’Angelo, Pescara, Italy; 5 SphingoTec GmbH, Hennigsdorf, Germany; 6 Department of Internal Medicine, Skåne University Hospital, Malmö, Sweden; 7 National Heart and Lung Institute, Imperial College London, London, UK

**Keywords:** Biomarkers, Cardiology, Endocrinology, Medical research, Diseases, Cardiovascular diseases

## Abstract

Postural orthostatic tachycardia syndrome (POTS) is a cardiovascular autonomic disorder with poorly understood etiology and underlying pathophysiology. Since cardiovascular morbidity has been linked to growth hormone (GH), we studied GH levels in patients with POTS. We conducted an age-sex-matched case–control study in patients with POTS (age 31 ± 9 years; n = 42) and healthy controls (32 ± 9 years; n = 46). Plasma GH levels were measured using high-sensitivity chemiluminescence sandwich immunoassay. The burden of orthostatic intolerance symptoms was assessed by the Orthostatic Hypotension Questionnaire (OHQ), consisting of a symptom assessment scale (OHSA) and a daily activity scale (OHDAS). POTS patients had significantly higher composite OHQ score than controls, more symptoms and less activity. Supine heart rate and diastolic blood pressure (BP), but not systolic BP, were significantly higher in POTS. Median plasma GH levels were significantly lower in POTS (0.53 ng/mL) than controls (2.33 ng/mL, p = 0.04). GH levels were inversely related to OHDAS in POTS and supine systolic BP in POTS and controls, but not heart rate neither group. POTS is associated with lower GH levels. Impairment of daily life activities is inversely related with GH in POTS. A higher supine diastolic BP is inversely associated with GH levels in POTS and healthy individuals.

## Introduction

Postural orthostatic tachycardia syndrome (POTS) is a cardiovascular autonomic disorder occurring predominantly in young women. POTS is characterized by an excessive heart rate increase when assuming upright posture accompanied by symptoms of orthostatic intolerance^1^. However, many POTS patients suffer from a wide array of non-specific ailments such as deconditioning, headache, dyspnea, muscle fatigue and cognitive impairment^1^. The pathophysiology of POTS has not been fully established but is likely to be multifactorial.

Different phenotypes of POTS have been identified, e.g. neuropathic POTS and hyperadrenergic POTS^2^. Likewise, POTS can be physiologically classified based on patterns of peripheral blood flow and peripheral arterial resistance into low-flow POTS, normal-flow POTS and high-flow POTS^3^. This variety of phenotypes suggests that POTS is a heterogenous syndrome with many manifestations sharing common pathophysiological mechanisms.

Human growth hormone (GH) is a peptide hormone secreted from the anterior hypophysis. GH plays a vital role in stimulating growth and development and maintenance of the cardiovascular system. Both up- and down-regulation of GH have been linked to increased cardiovascular morbidity and mortality. Upregulation of GH may cause hypertension, arrhythmias, diabetes mellitus, concentric cardiac hypertrophy, coronary artery disease, chronic heart failure, and stroke^4^, whereas downregulation of GH is characterized by dyslipidemia, insulin resistance, endothelial dysfunction, vascular atherosclerosis, a decrease in cardiac mass together with a post-exertional impairment of systolic function^5^. A possible explanation may be that subjects demonstrating GH level alterations have higher prevalence of hypertension, diabetes and cardiomyopathy^6,7^.

Previous evidence based on high-throughput proteomics analysis by proximity extension assay (PEA) technique suggested that levels of circulating GH might also differ between patients with POTS and other possible autonomic disturbances with a history of syncope but normal response to tilt testing^8^. Although targeted proteomics is an ideal approach for establishing biomarker signatures in various clinical settings, dedicated detection methods and adequately matched control populations are key for external validation of PEA-guided biomarker discovery.

Accordingly, we aimed to investigate circulating GH levels in POTS versus age- and sex-matched healthy control group from the same geographical location by high-sensitivity chemiluminescence sandwich immunoassay for plasma GH detection.

## Methods

The data that support the findings of this study are available from the authors upon reasonable request.

### Study design and population

The present study is based on the Syncope Study of Unselected Population in Malmö (SYSTEMA) cohort, with over 2200 patients investigated for syncope and severe orthostatic intolerance at the Skåne University Hospital in Malmö, Sweden between 2008 and 2019. Details of the SYSTEMA cohort are described elsewhere^9^. The SYSTEMA study protocol consists of cardiovascular autonomic testing including head-up tilt (HUT) testing with continuous hemodynamic monitoring, as well as other cardiac tests including ambulatory electrocardiogram (ECG) or 24-h ambulatory BP monitoring, when appropriate.

Forty-two consecutive POTS patients with heart rate increase of ≥ 30 bpm during HUT and chronic symptoms for ≥ 6 months were selected from SYSTEMA cohort^1^. Forty-six controls were recruited through personal invitation, among healthy medical students, Skåne University Hospital staff and younger participants of parallel population-based epidemiological programs in Malmö, Sweden. Controls had no history of syncope, orthostatic intolerance or endocrine disease. This part of the SYSTEMA project was approved by The Regional Ethical Review Board of Lund University, Sweden in a separate decision (2017/295). All participants, both POTS and controls, were examined at the Clinical Research Unit of Dept. of Internal Medicine, Skåne University Hospital, Malmö, Sweden.

All participants, i.e. POTS patients and healthy controls, were examined in a dedicated research facility. The study participants performed an active standing test, with 10-min rest in the supine position prior to standing. Blood pressure and heart rate were measured twice in the supine position by a validated automated oscillometer device (Omron, Kyoto, Japan), and then after 1, 3, 5, and 10 min of standing. An average of two measurements in the supine position was used for group comparisons. For orthostatic heart rate increase, difference between standing heart rate after 3 min and supine heart rate was calculated. All cardiovascular pharmacological agents such as beta-blockers, ivabradine, midodrine and droxidopa, were discontinued 72 h prior to examination. POTS diagnosis established during previous tilt-testing and characteristic symptoms were considered valid for POTS diagnosis in patients with pronounced orthostatic intolerance symptoms who were unable to abstain from medication or non-pharmacological measures (such as increased fluid intake or compression garments) that lead to improved hemodynamic responses on orthostatic stress and active standing.

### Orthostatic hypotension questionnaire (OHQ)

All study participants completed an adapted questionnaire based on the validated Orthostatic Hypotension Questionnaire (OHQ) consisting of two components: the six-item symptoms assessment scale (OHSA), and a four-item daily activity scale (OHDAS) to assess the burden of symptoms^10^.

Following symptoms are included in the OHSA: dizziness, problems with vision, generalized weakness, fatigue, trouble concentrating and head/neck discomfort. The participants were asked to rate each item from zero (none) to ten (worst possible) based on the severity of their symptoms during the past week.

The OHDAS includes questions related to how participants' orthostatic intolerance affects their daily life, e.g. activities that require standing/walking for a short/long time. Participants were asked to rate each item from zero (no interference) to ten (total interference), based on how much the activity has been interfered with during the past week.

### Growth hormone analysis

Study participants were asked to fast overnight and refrain from smoking, since both factors are known to influence the GH levels^11,12^. Ethylenediaminetetraacetic acid (EDTA) plasma samples were collected in the supine position and frozen at − 80 °C. GH levels were assessed using high-sensitivity chemiluminescence sandwich immunoassay for plasma GH detection (SphingoTec GmbH, Hennigsdorf, Germany).

The assay consists of two antigen-specific monoclonal antibodies, derived against recombinant 22 kDa growth hormone (somatropin) (NIBSC code 98/574, National Institute for Biological Standards and Control, Hertfordshire, UK) in a microtiter plate format. Both monoclonal antibodies bind to the antigen Vascular-Risk Human Growth Hormone (vr-hGH) at two individual epitopes. During incubation the vr-hGH interacts with the luminescent tracer antibody and capture antibody in the plasma. Upon indirect binding of the tracer antibody to the microtiter plate, the tracer remnant is washed away, and the level of captured tracer antibody can be analyzed using a luminometer.

The obtained luminescence signal is directly proportional to the level of vr-hGH in the plasma sample. The lower detection limit is 0.001 ng/mL and the functional assay sensitivity is 0.004 ng/mL, with an inter-assay coefficient of variation of 20%. Growth hormone is stable for 7 days in EDTA plasma at 2–35 °C and up to five freeze thaw cycles. Further details on the method are described elsewhere^13^.

### Statistical analysis

Between-groups comparison was performed using standard statistical tests, ANOVA for continuous variables and Pearson’s Chi-square test for categorical variables. Baseline characteristics of the study population were reported as mean and standard deviation or median and interquartile range, as appropriate, for continuous variables, and as counts and percentages for categorical variables. Growth hormone values were log-transformed and standardized before the group comparison to transform skewed data to approximately conform to normality. The relationships among GH levels, hemodynamic data and symptoms of orthostatic intolerance by OHQ were explored by linear regression analysis. Data were analyzed using SPSS software version 26 (SPSS, Chicago, IL, USA). P-value of < 0.05 was considered statistically significant.

### Ethical approval

The SYSTEMA project was approved by the Regional Ethical Committee in Lund, Sweden (82/2008), and all study participants provided informed written consent. All procedures were carried out in line with relevant current guidelines and regulations.

## Results

Characteristics of the study population are displayed in Table 1. Supine heart rate and supine diastolic BP were significantly higher in POTS patients compared with controls (69.0 ± 11.1 bpm vs. 63.3 ± 10.8 bpm, p = 0.02; and 72.9 ± 9.1 mmHg vs. 69.0 ± 8.5 mmHg, p = 0.04). Heart rate after 3 min of standing was also significantly increased in POTS patients (96.5 ± 16.0 vs. 83.1 ± 13.6, p < 0.001). However, we did not observe any significant difference in supine systolic BP (116.6 ± 13.3 mmHg vs. 115.2 ± 10.0 mmHg, p = 0.60) between the groups.

**Table 1:** Baseline characteristics and intergroup comparison between POTS and healthy controls.

	POTS (n = 42)	Controls (n = 46)	P-value
Mean age, years (age range, years)	31 ± 9 (19–62)	32 ± 9 (18–59)	0.54
Female, n (%)	36 (86)	35 (76)	0.25
SBP supine, mmHg	116.6 ± 13.3	115.2 ± 10.0	0.60
DBP supine, mmHg	72.9 ± 9.1	69.0 ± 8.5	0.04
HR supine, bpm	69.0 ± 11.1	63.3 ± 10.8	0.02
HR 3 min standing, bpm	96.5 ± 16.0	83.1 ± 13.6	< 0.001
OHQ score	60.0 ± 18.6	4.2 ± 7.5	< 0.001
OHSA score	36.2 ± 10.0	3.6 ± 6.4	< 0.001
OHDAS score	23.8 ± 9.7	0.6 ± 1.3	< 0.001

DBP: diastolic blood pressure; HR: heart rate; OHDAS: orthostatic hypotension daily activities; OHQ: Orthostatic Hypotension Questionnaire composite score; OHSA: Orthostatic Hypotension Symptom Assessment; POTS: postural orthostatic tachycardia syndrome; SBP: systolic blood pressure.

### POTS and OHQ score

POTS patients had significantly higher composite OHQ score than controls (60.0 ± 18.6 vs. 4.2 ± 7.5, p < 0.001), as well as OHSA (36.2 ± 10.0 vs. 3.6 ± 6.4, p < 0.001) and OHDAS scores (23.8 ± 9.7 vs. 0.6 ± 1.3, p < 0.001).

### Plasma levels of growth hormone in POTS

**Figure 1:**
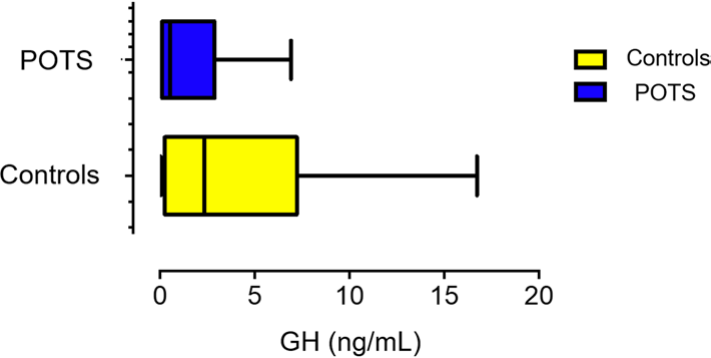
Levels of growth hormone in POTS versus healthy controls. GH, growth hormone; POTS, postural orthostatic tachycardia syndrome.

**Figure 2:**
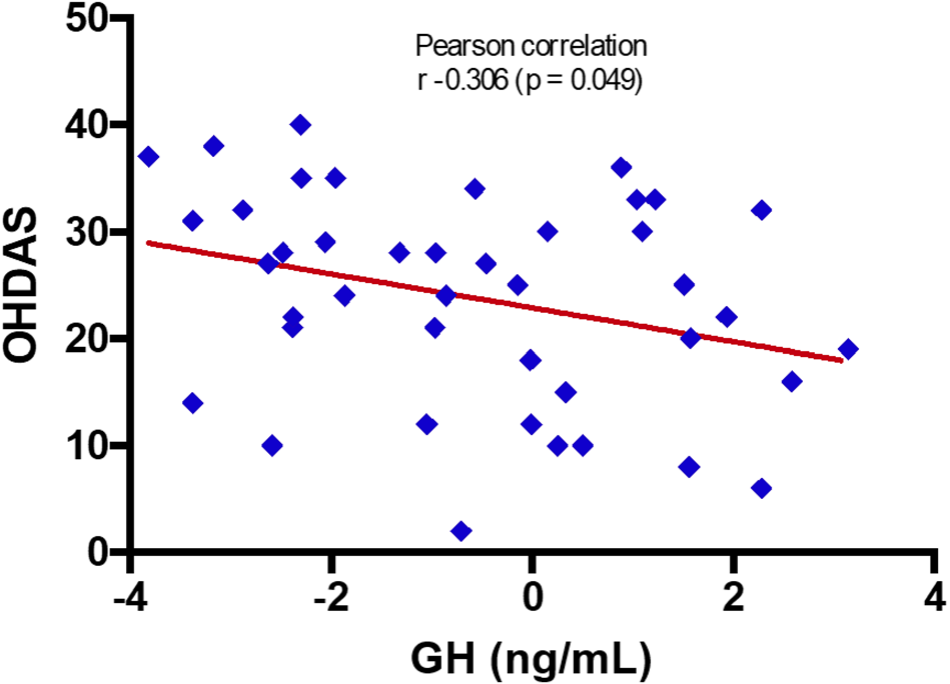
Correlation between circulating growth hormone levels and OHDAS score. GH, growth hormone; OHDAS, OH daily activities.

Plasma levels of GH were significantly lower in POTS patients compared with controls (median 0.53 ng/mL, IQR, 0.10–2.83 vs. 2.33 ng/mL, IQR, 0.26–7.2, p = 0.04, Fig. 1), although the GH levels were still within the normal range. Moreover, we found that levels of GH were inversely related to OHDAS (p = 0.049) among POTS patients (Fig. 2). However, OHQ and OHSA scores were not significantly associated with GH levels in POTS group (p = 0.37 and p = 0.84), respectively (not illustrated). In addition, post-hoc analysis showed no relationship between POTS symptom duration (median 4 years, IQR: 2–11) and levels of GH (p = 0.134) (not illustrated).

**Figure 3:**
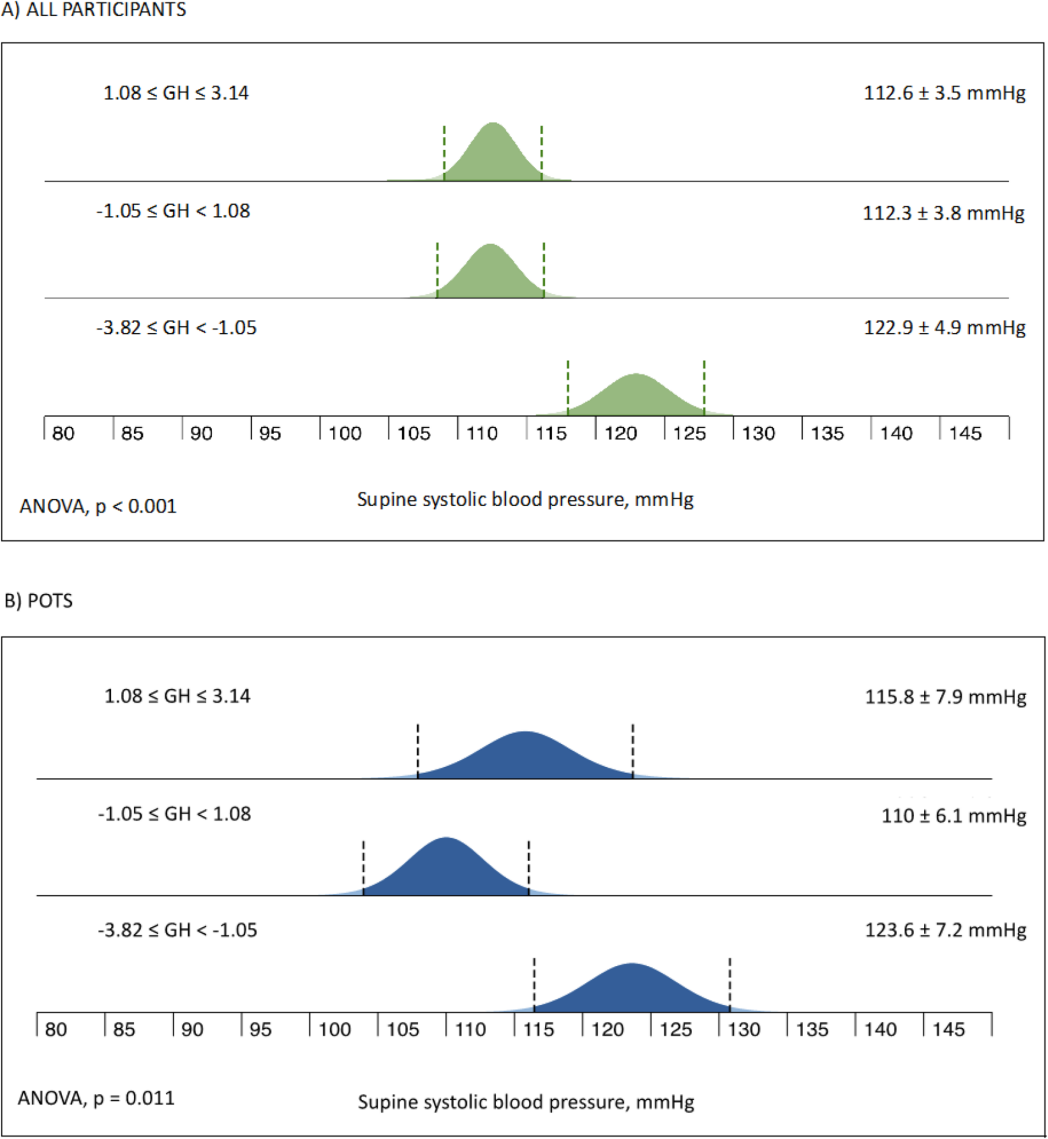
Supine systolic blood pressure by tertiles of circulating growth hormone levels in all participants (**A**, n = 84) and in POTS patients (**B**, n = 42). GH, growth hormone; POTS, postural orthostatic tachycardia syndrome.

Further, levels of GH were inversely associated with supine systolic BP (B = − 0.07, SE = 0.02; p < 0.001) in all participants, and in POTS (B = − 0.06, SE = 0.02, p = 0.004) but there was no association with heart rate, neither supine (p = 0.33) nor standing (p = 0.18), even when restricting the analysis to POTS subgroups (p = 0.53 and 0.25, respectively) (not illustrated). The association between lower GH and higher SBP demonstrated a distinct threshold level whereby SBP levels were significantly higher only in the lowest tertile of GH, both in all participants and POTS subgroup (Fig. 3).

## Discussion

In this study, we have demonstrated that patients with postural orthostatic tachycardia syndrome have lower levels of growth hormone. The lower levels of GH were associated with the overall severity of daily activity impairment among POTS patients, but not with the general and less specific symptoms such as weakness or problems with concentration.

Previous studies have shown that POTS patients have impaired quality of life^14,15^. Although it has been demonstrated that individuals with GH deficiency and compromised quality of life may benefit from GH replacement therapy^16^, the relationship between quality of life and GH deficiency in POTS is complex and warrants in-depth research.

Promising results have been reported about the beneficial effects of GH replacement therapy on the cardiovascular system in individuals with GH deficiency. It has been shown that GH replacement significantly improves various cardiovascular risk parameters, e.g. lipid-profile and glucose metabolism, with other possible pleiotropic cardioprotective effects^17–19^. However, potential clinical utility and efficacy of GH replacement therapy on symptom burden of POTS patients and its impact on cardiovascular risk profile is largely unknown.

The hypothalamus secretes growth hormone-releasing hormone (GHRH), which stimulates GH synthesis and secretion to a large extent, while somatostatin inhibits the GH secretion from the anterior hypophysis^20^. Previous studies have shown that low levels of GH are associated with an excessive autonomic sympathetic activity. In GH deficiency, the levels of GHRH are increased, prompting the authors to assume that the effect of low GH may be attributed to a positive feedback loop between GHRH and norepinephrine. Since GH release is blocked in GH deficiency, a rise in norepinephrine occurs. This efflux of norepinephrine may in turn enhance production of GHRH and, thus, release of GH^21^. However, this may not be the case in POTS.

POTS is associated with abnormal sympathetic hyperactivity. Norepinephrine metabolism is described as abnormal in hyperadrenergic POTS with increased levels of plasma norepinephrine^1,22^. The possible effect of GH on the autonomic nervous system may be more evident in some individuals, thus, potentially leading to a greater predisposition to POTS-related symptoms. Although at the current stage of understanding, the relative place of GH and norepinephrine in the pathophysiological cascade of POTS is not yet established.

Moreover, exercise is a strong stimulus of GH secretion^23^. Reduced exercise capacity and physical deconditioning occurs in both patients with POTS and GH deficiency^1,21^, however, whether GH levels are related to mechanism of POTS is yet unknown. It may be speculated that the observed lower levels of GH in the present study may be a consequence of POTS-induced physical inactivity. Interestingly, evidence has demonstrated that deconditioning in POTS may be associated with reduced left ventricular mass (LVM), and after starting physical training the LVM increased in individuals with POTS to the same size as in controls^24,25^. GH deficiency is also associated with reduced LVM^26^, and studies have demonstrated favorable effects of physical exercise, such as aerobic training in patients with either POTS or GH deficiency. Although the advantages of exercise in GH deficiency may resemble the effects of GH replacement therapy, previous results indicate that the positive effects of physical exercise are not additive to GH therapy^27^.

GH exerts also diabetogenic and anti-insulin properties, partly due to its indirect effects via insulin-like growth factor 1 (IGF-1), which has glucose-lowering effects similar to insulin^28^. Consequently, it may be hypothesized that reduced GH levels may result in enhanced insulin action^29^, conferring a higher risk of prediabetes in a subset of patients with POTS.

A previous study has reported elevated plasma levels of GH in POTS, by using a proteomics approach and analyzing simultaneously over 90 cardiovascular plasma biomarkers in one plasma sample^8^. In our study, we observed lower levels of GH in POTS patients, by using a highly specific immunoassay method. These contradictory findings may be partly explained by the techniques used to analyze GH, since the proteomics technique targeted a wide spectrum of different biomarkers, thus obtaining relative GH levels, whereas our method was targeted at absolute quantification of GH levels. However, the contradictory findings may also be partly explained by partly different study groups. The previous study^8^ included patients with a normal hemodynamic test as the control group, unlike including healthy subjects from the population, which was the case in our present study. Also, the timing of the measurements in the stage of disease, as well as the clinically insignificant variations in GH level that were within normal ranges may further explain the conflicting results.

Moreover, we found that lower levels of GH were associated with higher supine systolic blood pressure in both POTS and healthy individuals. Interestingly, alterations in heart rate were not associated with GH levels. Thus, lower GH levels may paradoxically indicate higher supine BP but reduced orthostatic intolerance, suggesting that alternative mechanisms other than hemodynamic, may be involved in POTS-symptom generation mediated by lower GH levels.

Another previous study reported that patients with GH deficiency also have increased supine systolic blood pressure in comparison with healthy controls, whereas no difference was observed in heart rate^30^. Notably, the study found that individuals with GH deficiency have an abnormal blood pressure response during orthostatic stress, possibly attributed to structural changes of the aorta and carotid artery, which are responsible for the baroreflex control of BP.

Another possible explanation of our observations may be an upsurge in norepinephrine. Since secretion of GH is regulated by norepinephrine, low levels of GH may contribute to elevated SBP by stimulating the sympathetic nervous system. Ultimately, the underlying pathophysiological link needs further investigation in larger and independent populations.

### Strengths and limitations

With the biology of POTS incompletely explored, our study supports the association between GH levels and POTS. To the best of our knowledge, this is the first study aimed at directly measuring the levels of GH in POTS. The discordant findings observed in this study with regard to previous proteomics evidence with PEA technology needs further verification in independent cohorts. A biomarker-driven approach for phenotyping POTS may improve the understanding of its pathophysiology.

Although GH levels were associated with impairment of daily life activities and higher supine systolic blood pressure, however, whether there exists a causal relationship between the two events remains to be investigated. It is also unclear if exercise and growth hormone supplementation may potentially influence our observed findings. Moreover, the study sample size of our study was relatively small. Likewise, the observed levels of growth hormone in POTS and controls were in fact within normal ranges, e.g. in adult males 0.4 to 10 ng/mL, and in adult females 1 to 14 ng/mL.

As far as possible clinical implications of this explorative study are concerned, the present results do not yet have any direct therapeutic or diagnostic implications in subjects with POTS. In the long term and for possible clinical relevance, the role of GH should be tested not only among POTS, but also in similar diseases of cardiovascular autonomic function. It may be speculated that measurement of GH in plasma may be useful in POTS to determine the underlying neuroendocrine alterations and aid in clinical decision-making regarding a possible pharmacological intervention or substitution. Considering that patients with POTS have pronounced central nervous system symptomatology, it would be of interest to determine whether the hormonal dysregulation is at the hypothalamic level rather than the pituitary. Future studies should be aimed at assessing the hormonal profile in POTS, including the hypothalamic–pituitary–adrenal axis, which is involved in fluid and salt conservation, as well as measurement of insulin-like growth factor 1 (IGF-1).

## Conclusions

The multifaceted nature of postural orthostatic tachycardia syndrome poses many diagnostic and therapeutic challenges. Our study adds to the understanding of POTS and shows that patients with POTS have significantly lower plasma levels of circulating growth hormone. Impairment of daily life activities is inversely related with growth hormone levels in POTS. A higher supine diastolic blood pressure is inversely associated with growth hormone levels in both POTS and healthy individuals. Further research is needed to confirm these findings in larger and independent populations.

## Data Availability

The datasets analyzed during the current study are available from the corresponding author on reasonable request.
